# Successful treatment in a child with enthesitis-related arthritis involving the sternoclavicular joint: a case report

**DOI:** 10.1186/s12887-019-1770-6

**Published:** 2019-10-23

**Authors:** Po-Yu Huang, Ling-Sai Chang, Mindy Ming-Huey Guo, Ho-Chang Kuo

**Affiliations:** 1 Department of Traditional Chinese Medicine, Kaohsiung Chang Gung Memorial Hospital and Chang Gung University College of Medicine, #123 Da-Pei Road, Niaosong District, Kaohsiung, 83301 Taiwan; 2 Department of Pediatrics, Kaohsiung Chang Gung Memorial Hospital and Chang Gung University College of Medicine, #123 Da-Pei Road, Niaosong District, Kaohsiung, 83301 Taiwan

**Keywords:** Etanercept, Juvenile idiopathic arthritis, Sternoclavicular joint, Ultrasonography

## Abstract

**Background:**

Although the sternoclavicular joint (SCJ) may be involved in ankylosing spondylitis, rheumatic arthritis, and Behçet’s disease and participates in the systemic inflammatory process of arthritis, it is often neglected during routine rheumatologic clinical examinations. To the best of our knowledge, this is the first study to report etanercept treatment in juvenile idiopathic arthritis (JIA) with SCJ involvement.

**Case presentation:**

In this study, we describe an unusual case of a child with juvenile idiopathic arthritis with an initial presentation of sternoclavicular mass. The patient (age, 14 years 10 months) presented with an insidious onset atraumatic swelling of the left SCJ and complained of right hip and bilateral ankle tenderness without an apparent cause. Initial ultrasonography indicated a heterogeneous mass in the left SCJ, while computed tomography identified mild swelling of the left SCJ with a thickened synovial lining, mild bone erosion, and some turbid fluid. The patient ultimately underwent left SCJ arthrotomy, during which tapping of the SCJ revealed 2 cc of yellowish fluid, inflammation and necrosis of tissues within the SCJ. A clear yellow joint fluid was aspirated, and testing revealed a negative culture result. The patient was diagnosed with JIA. The joint tenderness improved and erythrocyte sedimentation rate decreased after administering anti-tumor necrosis factor etanercept. An additional ultrasonography demonstrated that the initial imaging findings have been resolved. At the end of a 2-year follow-up period, the patient was completely symptom-free.

**Conclusions:**

JIA with SCJ involvement is an uncommon presentation in adolescents. Etanercept may be a beneficial treatment for SCJ involvement in patients with JIA. The upper limbs showed no signs of limited range of motion during the follow-up period. Further studies are warranted to elucidate the efficacy of etanercept in JIA with sternoclavicular joint involvement.

## Background

Juvenile idiopathic arthritis (JIA) is a heterogeneous group of disorders. Enthesitis-related arthritis. (ERA) is a category of JIA, defined by the International League of Associations for Rheumatology (ILAR) [1]. This form of JIA is characterized by enthesitis and hip arthritis [2]. Sacroiliitis and low back pain (LBP) develop in the later stages of the disease. ERA demonstrates vast geographical differences, with a remarkably high prevalence (37.4%) in the Taiwanese population [3]. The etiology of JIA is hypothesized that a susceptible individual with distinct genetic background could develop an uncontrolled immune response towards a self-antigen on exposure to an uncertain stimulation [4]. The expansion of pathogenetic studies has increased our understanding of the immunopathogenesis. Depression of CD8+ suppressor T lymphocyte functions is one of the important mechanisms underlying clinically active JIA [5–7].

Sternoclavicular joint (SCJ) lesions are rare and commonly ignored as they are often painless or considered benign. Nevertheless, SCJ lesions have a number of diagnostic and therapeutic standards. Possible differential diagnoses of SCJ can include infective, neoplastic, rheumatological, degenerative, and idiopathic conditions [8]. The differential diagnosis of unilateral SCJ arthritis is sometimes challenging as it can include infective and inflammatory pathologies like sternal osteomyelitis, septic arthritis, and systemic arthritis of SCJ. A previous report described synovial cysts involving bilateral SCJs in a poly-articular JIA case [9]. Synovial proliferation and accumulation of synovial fluid may contribute to the development of a synovial joint cyst. In systemic arthritis, osteoarthritis is the most frequent cause of pain and swelling of the SCJ [8]. A strong association has been found between seronegative spondyloarthropathy and SCJ involvement [10]. Traditionally, spondyloarthritis has been differently classified in adults and children. Using the ILAR system for JIA, most childhood spondyloarthritis is classified as enthesitis-related arthritis [11]. A retrospective study analyzing Brazilian patients diagnosed with ankylosing spondylitis identified sternoclavicular involvement in 14.3% [12]. Juvenile spondyloarthritis has been associated with articular involvement of the lower limbs, but not sternoclavicular involvement. However, the SCJs may be one of the first joints to become involved in juvenile ankylosing spondylitis [13]. A previous case-control study reported 14 patients with juvenile spondyloarthritis accompanied with costosternal pain, six of which had reduced chest expansion [14]. Using ultrasound, another study detected greater synovitis and erosions of the involved SCJ in patients with rheumatoid arthritis [15]. Behçet’s disease may also involve the SCJs. In one study, two patients presented with destructive arthritis that involved the SCJ [16].

The following study details the case of a 14-year-old boy with a 3-month history of pain and swelling of the left SCJ. This report aims to present our experience with SCJ management in JIA.

## Case presentation

A 14-year-10-month-old Han Taiwanese boy was presented to the pediatric out-patient clinic with a 3-month history of swelling of the left clavicle. His family history included his father’s ankylosing spondylitis and his mother’s uveitis. Physical examination revealed mild tenderness. An erythematous immobile firm mass measuring approximately 2 × 2.5 cm was found overlying the left proximal clavicle, immediately lateral to the SCJ. Furthermore, he expressed pain when the clinician pressed down on his right hip and bilateral ankles. We did not perform modified Schober test in our patient because LBP was not obvious and he was a suspicious case of JIA.

The patient had right hip arthritis and bilateral Achilles enthesitis. White blood cells count was 7.8 × 10^3^ /μl, with 57% neutrophils and 32% lymphocytes (normal 3.9–10.6 × 10^3^ /μl; neutrophils 42–74%; lymphocytes 20–56%). Biological inflammatory syndrome was observed with elevated erythrocyte sedimentation rate (33 mm/hr.; normal < 17 mm/hr) and C-reactive protein (22.6 mg/l; normal < 5 mg/L). Human leucocyte antigen (HLA) typing was positive for B27. Meanwhile, uric acid, rheumatoid factor, complements C3 and C4, anti-nuclear antibody, and anti-double-strand DNA were all normal. Furthermore, his renal and liver function, urine analysis, and muscle enzyme tests results were all normal.

**Fig. 1:**
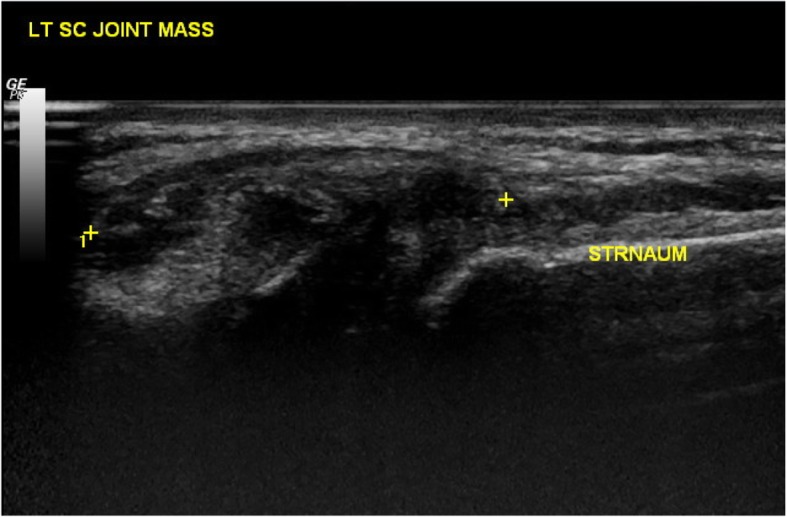
Ultrasonography of the left sternoclavicular joint shows a heterogeneous hypoechoic mass in a 14-year-10-month-old male patient with juvenile idiopathic arthritis

**Fig. 2:**
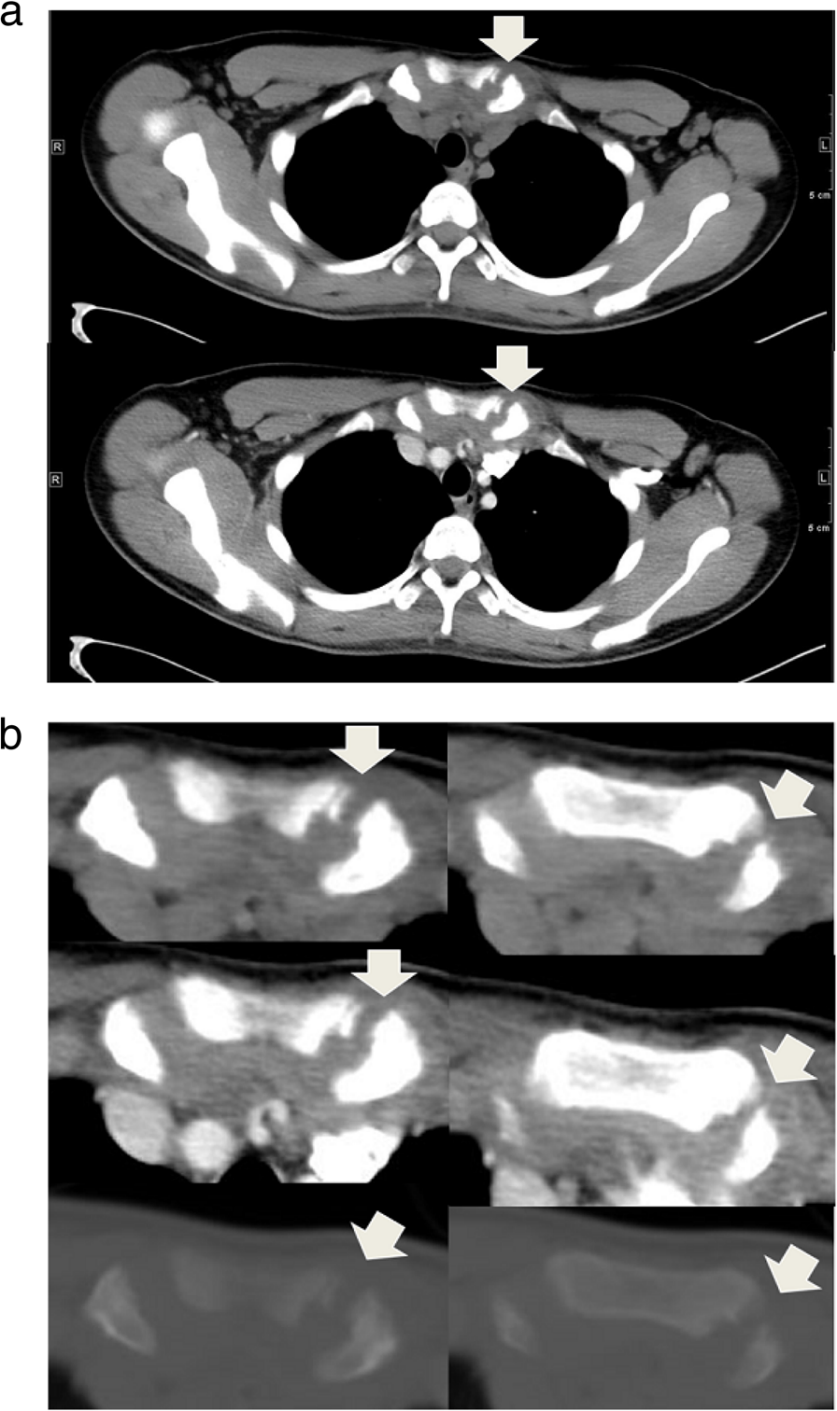
**a** Enhanced axial computed tomography scan of the sternoclavicular joints (SCJs) shows mild swelling of the left SCJ with thickened synovial lining and mild bone erosion, as well as some turbid fluid collection around the medial end of the clavicle (arrow). **b** Enhanced zoom-in computed tomography of the sternoclavicular joints (bone window, bottom)

**Fig. 3:**
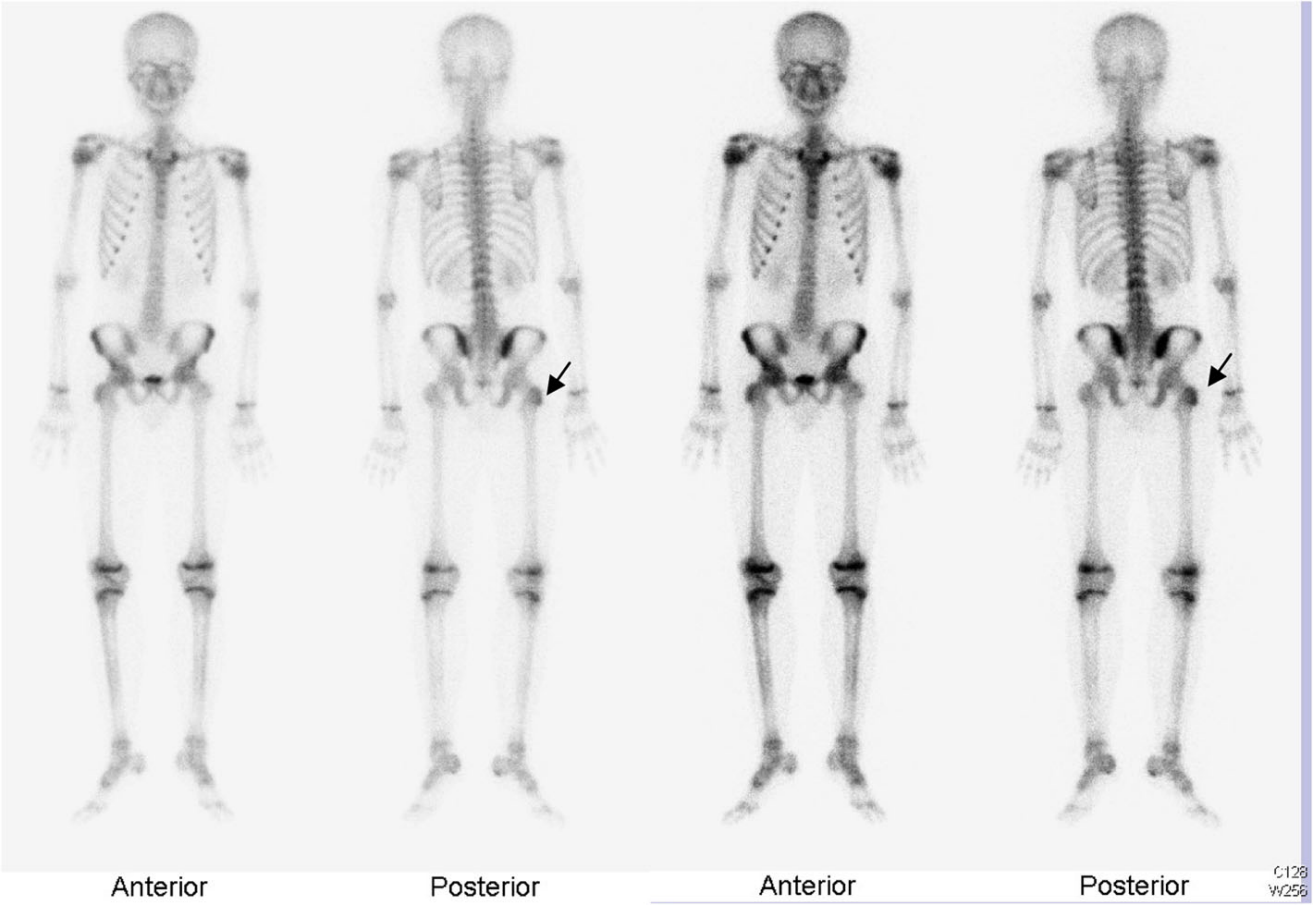
A bone scan reveals increased uptake of the right proximal femur at the trochanteric region, reflecting the patient with juvenile idiopathic arthritis in whom the bones were affected

The patient was initially treated empirically with oral augmentin (amoxicillin trihydrate + clavulanate potassium) and cefixime for 1 week, but did not respond favorably to the antibacterial treatment. We then treated the patient with naproxen (500 mg/day), a non-steroidal anti-inflammatory agent, and followed up. He claimed to have no history of trauma, fever, weight loss, loss of appetite, or intravenous drug abuse. We observed no skin lesions, such as acne or pustulosis palmaris et plantaris. Ultrasonography examination revealed a heterogeneous hypoechoic mass in the left SCJ (2 × 2.5 cm; Fig. 1), and he was hospitalized to our pediatric ward. A computed tomography (CT) scan of the chest further indicated a turbid collection at the left SCJ (Fig. 2). Bone window testing demonstrated irregular bone surfaces on the SCJ due to erosion, thus suggesting arthritis. Bone scintigraphy (MDP Tc-99 m) showed a focal area of increased uptake in the trochanteric region of the right proximal femur (Fig. 3).

We performed a left SCJ arthrotomy on the patient, in which the clavicular head was excised and the left SCJ was debrided. Due to suspicion of malignant infiltration, we performed a histopathological examination, but no malignant cells were observed. A histologic examination of the tissue exposed acute and chronic inflammatory cell infiltration. Aspirated joint fluid cultures were negative; therefore, diagnosis of infection could be excluded.

**Fig. 4:**
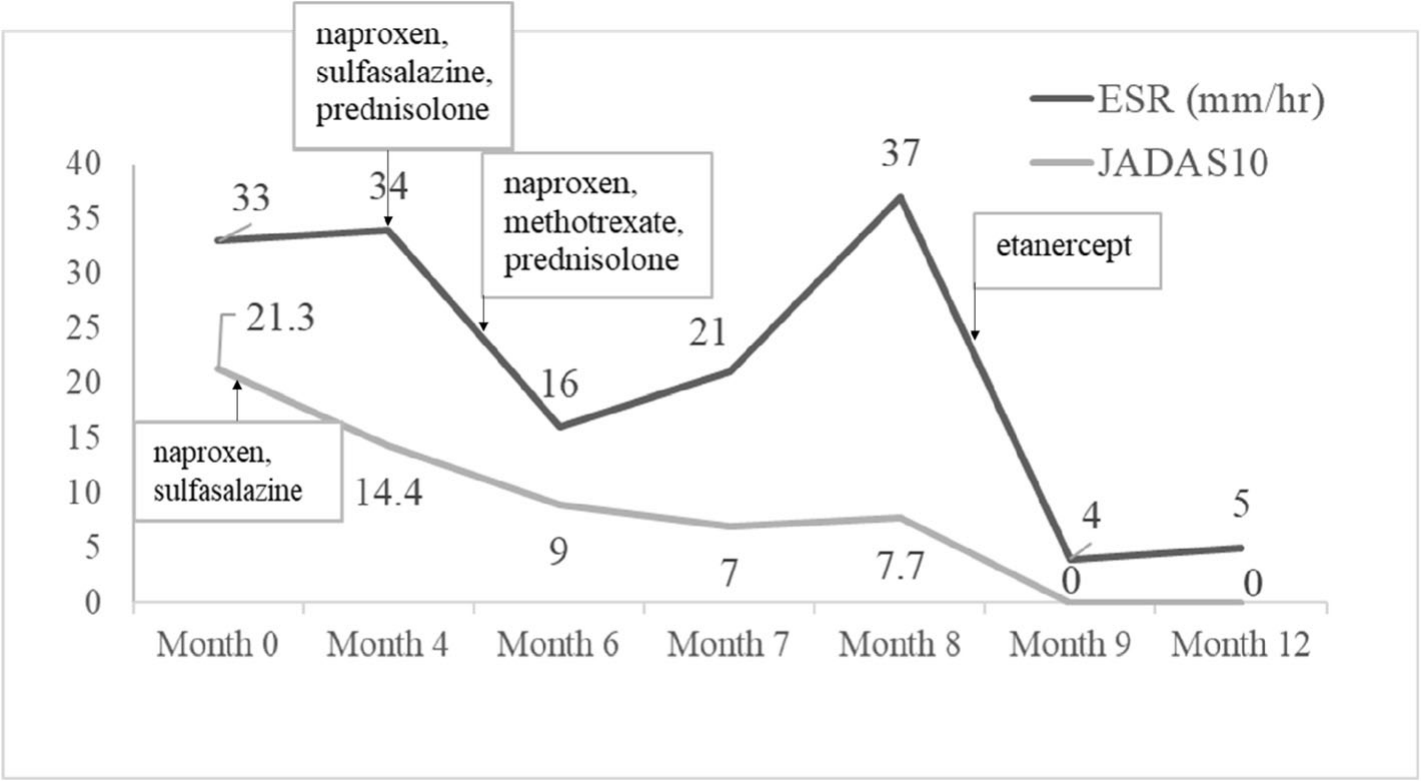
The erythrocyte sedimentation rate (ESR) and 10-joint Juvenile Arthritis Disease Activity Score (JADAS10) in a child with juvenile idiopathic arthritis during the observation period. The joint pain and swelling completely resolved in the second month of treatment with etanercept, and laboratory values improved in accordance with the clinical course of the patient

In view of the historical facts and clinico-radiological correlation, including CT findings, we made a working diagnosis of JIA of the medial end of the left clavicle and initiated JIA therapy. The onset of arthritis and enthesitis after 6 years of age in a boy with HLA-B27 and a family history allowed him to meet the strict diagnostic criteria of the JIA subtype enthesitis-related arthritis. Given that SCJ disorders are rare, there is a probability that many clinicians do not have substantial training or experience injecting the SCJ with corticosteroids [17]. This lack of experience, combined with a complex and frequently distorted SCJ anatomy, may result in poor injection accuracy. After surgical debridement, we prescribed a naproxen, sulfasalazine 1000 mg/day (4 months before prednisolone), and prednisolone 0.6 mg/kg/day combination for 4 weeks, followed by naproxen + oral methotrexate (MTX) 10 mg/m^2^/week + prednisolone for the next 3.5 months. Due to persistent high disease activity under salfasalazine, an alternate immunomodulator therapy MTX was selected. Etanercept has been indicated in the treatment of refractory JIA [18]. He achieved full remission from the JIA with etanercept 25 mg twice a week within 4 weeks (Fig. 4), and tolerated etanercept with good adherence. The patient reported complete cessation of pain and no longer needed analgesics, MTX, or prednisolone after 3 months. The aim of our treatment was to achieve maximum effectiveness and reduce the dosage of concomitant therapy because concomitant therapy substantially elevates the risk of side effects. A prospective study also revealed the effectiveness of etanercept therapy [19]. Furthermore, 38.3% of patients with JIA treated with etanercept in combination with any medication could completely discontinue these concomitant drugs. Our patient continued the etanercept treatment 25 mg twice a week for at least 2 years to maintain a stable condition without any relapse during the outpatient follow-up. We repeated the ultrasound of the left clavicle, which revealed complete healing of the initial findings after 13 months of etanercept treatment.

## Discussion and conclusions

In this case report, we described a 14-year-10-month-old male patient with JIA who received biologic treatments. This case suggests a possible relationship between JIA and SCJ involvement.

Anti-tumor necrosis factor is an effective treatment for patients with JIA resistant to non-steroidal anti-inflammatory drugs and disease-modifying anti-rheumatic drugs (DMARDs). There is evidence to support that Etanercept, one of the TNF-α inhibitors, improves JIA-associated symptoms and prevents flare [20, 21]. Additionally, Etanercept demonstrated its effectiveness in ERA [22, 23]. A meta-analysis concluded that etanercept is as effective as abatacept, adalimumab, and tocilizumab [24]. However, etanercept was not successfully discontinued in patients without JIA recurrence [25]. Patients treated with etanercept have a lower chance of infection compared to patients treated with infliximab [26]. Taiwanese children with JIA who received TNF inhibitors were not at higher risk of tuberculosis [27]. The reported adverse events of etanercept were hypersensitive joint reaction, skin rash, upper respiratory tract infection, monoarticular septic arthritis, headache, and fatigue [28, 29]. Currently, DMARDs and corticosteroids are largely used as bridge or adjunctive therapies [30]. The ERA category usually affects > 6-year-old boys and presents with enthesitis. The presence of hip or ankle arthritis and a family history of spondyloarthropathy or polyarticular joint involvement at onset are correlated with poor prognosis [31]. Current guidelines support the use of sulfasalazine to treat ERA [15].

The most important differential diagnosis is chronic non-bacterial osteitis (CNO). Synovitis, acne, pustulosis, hyperostosis, osteitis syndrome (SAPHO) describes the same disease entity. The bone scintigraphy result indicates a lesion in the femur. This makes CNO a probable diagnosis for this case. Magnetic resonance imaging figures were inconclusive, but the involvement of SCJ may be due to primary involvement of proximal end of clavicula with CNO. Therapeutic strategies for CNO include non-steroidal anti-inflammatory drugs (NSAIDs), which are often highly effective [32]. Etanercept could be effective in CNO. However, our patient responded poorly to NSAIDs. Furthermore, the strong family history with positive HLA-B27 suggested a case of ERA.

This is the first report of successful use of etanercept in the treatment of JIA with SCJ involvement. In general, physicians should pay attention to SCJ involvement in patients with JIA. Future prospective studies are warranted to testify whether adding etanercept to DMARDs can rescue SCJ involvement in JIA.

## Data Availability

Please contact the authors for all data requests.

## Abbreviations

Anti-TNF: Anti-tumor necrosis factor
CNO: Chronic non-bacterial osteitis
CT: Computed tomography
DMARDs: Disease-modifying anti-rheumatic drugs
ERA: Enthesitis-related arthritis
HLA: Human leucocyte antigen
ILAR: International League of Associations for Rheumatology
JIA: Juvenile idiopathic arthritis
LBP: Low back pain
MTX: Methotrexate
NSAIDs: Non-steroidal anti-inflammatory drugs
SCJ: Sternoclavicular joint
